# Serum tissue factor as a biomarker for renal clear cell carcinoma

**DOI:** 10.1590/S1677-5538.IBJU.2017.0007

**Published:** 2018

**Authors:** Daniel D’Oliveira Silva, Jorge A. P. Noronha, Bartira E. Pinheiro da Costa, Pedro Caetano Edler Zandona, Gustavo F. Carvalhal

**Affiliations:** 1 Departamentos de Urologia, Pontifícia Universidade Católica, Porto Alegre, RS, Brasil; 2 Instituto de Pesquisas Biomédicas (BEP), Pontifícia Universidade Católica, Porto Alegre, RS, Brasil

**Keywords:** Kidney Neoplasms, Thromboplastin, Biomarkers

## Abstract

**Purpose:**

to determine the usefulness of serum TF as a potential marker for patients with clear cell RCC.

**Materials and Methods:**

prospective study of 30 patients with clear cell RCC submitted to nephrectomy and 16 controls without clear cell RCC treated surgically for other conditions. TF is a endothelium marker that was correlated with worse prognosis in a variety of solid tumors including RCC. Serum TF was collected before surgery at the operating room and in the postoperative setting after at least four weeks. Serum samples were analyzed with a commercial ELISA kit for human TF (R&D Systems^®^).

**Results:**

Mean preoperative serum TF levels in clear cell RCC patients and in controls were 66.8 pg/dL and 28.4 pg/dL, respectively (p<0.001). Mean postoperative serum TF levels in clear cell RCC patients were 26.3 pg/dL. In all patients with clear cell RCC postoperative serum levels of TF were lower, with a mean reduction of 41.6 pg/dL in the postoperative setting (p<0.001). Linear regression revealed that tumor size was correlated with the postoperative reduction of serum TF levels (p=0.037).

**Conclusions:**

We have shown a 3-fold reduction in the median preoperative serum levels of TF in patients with clear cell RCC after surgery. We have also shown a difference of the same magnitude in the serum levels of TF compared with those of a control group of patients with benign diseases. TF appears to be a useful serum marker for the presence of clear cell RCC. Further studies are needed to validate these findings.

## INTRODUCTION

Clear cell renal cell carcinoma (RCC) corresponds to 85% of all renal malignancies, with an estimated 66.000 new cases in the U.S. in 2016 (1). In 2012, there were about 142.000 deaths due to kidney neoplasms, representing the 16^th^ most common cause of cancer specific mortality (2). In spite of the significant increase in the diagnosis of incidental RCCs, the expected reduction in mortality rates did not occur, since roughly 30% of the patients with RCCs are already metastatic at presentation (3). In fact, mortality rates of renal malignancies have increased in underdeveloped and in developing countries in recent years (4).

The search for new diagnostic and prognostic biomarkers in clear cell RCC has evolved slowly over the years. Many tissue biomarkers have been studied, with mixed results as either prognostic or predictive tools: carbonic anhydrase IX, B7-H1, survivin, PD-1, Epcam/ksa, IGF-1, VEGF, EphA2, and Skp2 (5-13). More recently, some genes were associated with the presence of CCR. Among these, the most frequently mutated were: PBRM1, that is part of the chromatin remodeling complex, BAP1 and SETd2, which are histones also relevant to this process (14, 15). So far, no serum biomarker has proven clinically useful in clear cell RCC.

Tissue factor (TF) is a transmembrane protein responsible for triggering the extrinsic coagulation pathway (16). An increased TF expression was correlated with worse outcomes in various tumors from organs such as pancreas, ovary, lung, breast, prostate, colon and central nervous system (17). Serum TF levels were tested as possible tumor markers in ovarian and pancreatic cancers, with promising results (18, 19). We have previously reported that an increased immunohistochemical expression of TF in Wilms tumors (20) and in clear cell RCC (21) was an adverse prognostic factor, correlated with decreased overall and cancer-specific survival. The present study aims to determine whether serum levels of TF are useful as biomarkers in clear cell RCC.

## MATERIALS AND METHODS

A prospective study was carried out in patients with renal tumors candidates for either radical or partial nephrectomy at a university hospital. From August 2014 to July 2016, 43 patients were included in the study, and were tested preoperatively for TF. Of these, 11 patients who did not present with the diagnosis of clear cell RCC in the pathologic exam (08 benign lesions, 02 chromophobic carcinomas, 01 papillary carcinoma) were excluded. In 32 patients with confirmed diagnosis of clear cell RCC, a second blood collection for TF was performed after at least four weeks from the date of surgery. Additionally, we tested for TF in the preoperative setting the serum of 16 controls who were operated with diverse diagnosis. This heterogenous group was composed by patients with prostate cancer (1), benign renal cysts (2), oncocytoma (1), benign prostatic hyperplasia (7), non-functional kidneys (2), ureteral stones (2), and renal stone (1). Controls were operated by the same Urology team, and showed no evidence of RCC in imaging exams of the upper urinary tract and in pathology reports. Postoperative samples were lost in two patients with clear cell RCC, who were excluded from the study. Our final study Group thus consisted of 30 patients with clear cell RCC and 16 controls with other diseases. The clinical and pathologic variables studied included: age at diagnosis, gender, presence of arterial hypertension, history of smoking, Fuhrman’s grade, TNM stage (2010), presence of tumor necrosis, size of the renal lesion, preoperative and postoperative serum levels of TF.

### TF-Enzyme-Linked Immunosorbent Assay (ELISA)

Serum concentrations of FT were quantified by ELISA using the Human Coagulation Factor III/Tissue Factor Immunoassay-Quantikine^®^ ELISA kit (Catalog Number DCF300) purchased from R&D (California-USA). Samples of 5mL of blood were collected in tubes containing EDTA before and after at least four weeks of surgery. Within 30 minutes after collection, serum was separated by centrifugation at 1000 x g for 15 minutes. Aspirated, the serum was transferred to 600μL tubes and stored in a freezer at -80°C. When the sample collection was complete, testing was initiated, following the manufacturer’s protocol. In the 96-well polystyrene plates with monoclonal antibody specific for coagulation factor III, the standard curve (concentrations 7.8 to 500pg/mL) and the samples were added. After 2 hours of incubation at room temperature, the plates were aspirated and washed 3 times. The same procedures were repeated for addition of the conjugate. Addition of the substrate was incubated for 30 minutes without shaking and protected from light. At its end 50μl of sulfuric acid solution was added to suspend the reaction. The optical density of the samples was obtained by reading the plate in Zenyth^®^ 340R (Made in Austria) spectrophotometer with a 450nm filter with 570nm wavelength correction. Tissue factor concentrations of the samples were calculated with respect to the reading of the standards and the correlation between the points of the standard curve (r2=0.9963). At the end the result was multiplied by 2 due to the dilution of the samples recommended by the R&D manufacturer. The tests were performed at the Laboratory of Nephrology of the Biomedical Research Institute of PUCRS.

### Statistical Analysis and Ethics

Continuous variables were described as means and standard deviations. For asymmetric data we used medians and minimum-maximum intervals. Categorical data were expressed using counts and percentages. We used Student’s t-test for the comparison of continuous variables and Fisher’s exact test for the comparison of variables with categorical data. A linear regression was used to identify the potential association of the reduction of serum TF with tumor size as with other quantitative variables. The values were expressed as r (Pearson’s coefficient) and b (angular coefficient). For the association of categorical variables, covariance analysis with negative values and standard deviation was used. The Statistical Package for Social Sciences program (SPSS, v. 22.0) was used for data analysis. The study protocol was reviewed and approved by the research ethics committee of the university hospital, protocol number 33687414.5.0000.5336, and all patients signed a free informed consent form when included in the study.

## RESULTS

The clinical and pathologic variables are shown in Table-1. There was a predominance of men in the sample (n=18, 60.1%), and the mean age was 58.8 years (31-91 years). Mean tumor size was 5.53cm (0.4-19.0cm). Regarding Fuhrman’s classification, 4 (13.3%) patients were grade 1, 20 (66.6%) were grade 2, 6 (20%) were grade 3, and 0 (0.0%) were grade 4. The pathologic stage was TNM I in 22 (73.3%); TNM II in 02 (6.6%), TNM III in three (10%), and TNM IV in three (10%). There were no statistically significant differences between Groups regarding age, sex, smoking and hypertension.

**Table 1:** Patients characteristics.

Characteristics		CCR	CONTROLS	p
		n=30	n=16	
Age, years		61.1±10.1	57.5±12.9	0.950^[a]^
Male sex, n (%)		18 (60.0)	12 (75.0)	0.257^[b]^
Tumour size, cm		5.52±2.5	-	-
Hypertension		17(56.6)	4(25.0)	0.060
**Fuhrman grade, n (%)**				-
	1	4 (13.3)	-	
	2	20(66.6)		
	3	6 (20.0)	-	
	4	0 (0.0)	-	
**TNM stage, n (%)**				-
	I	22 (73.3)	_-_	-
	II	2 (6.6)	-	
	III	3 (10.0)	-	
	IV or more	3 (10.0)	-	

Data are presented as mean ± standard deviation or counts (%).

**TNM** = TNM staging classification for renal cell carcinoma.

**p** = statistical significance; **[a]** = Student’s t test; **[b]** = Fisher's exact test.

**Figure 1:**
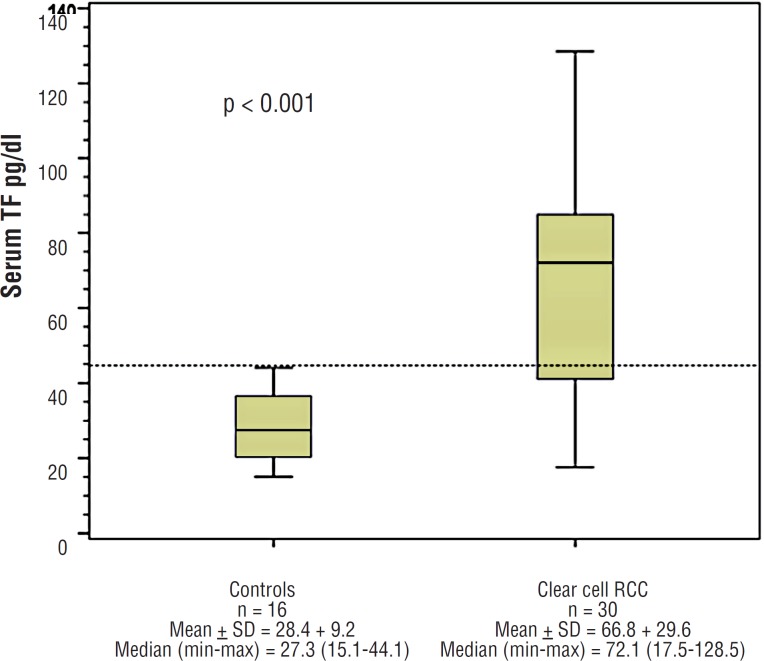
serum TF in Controls and in Clear Cell RCC.

The mean pre-operative serum TF in patients with clear cell RCC was 66.8pg/dL whereas in the control Group it was 28.4pg/dL (p <0.001). Figure-1 shows a box-plot representation of the mean and median values of TF in serum, both in renal cancer and in controls.

**Figure 2:**
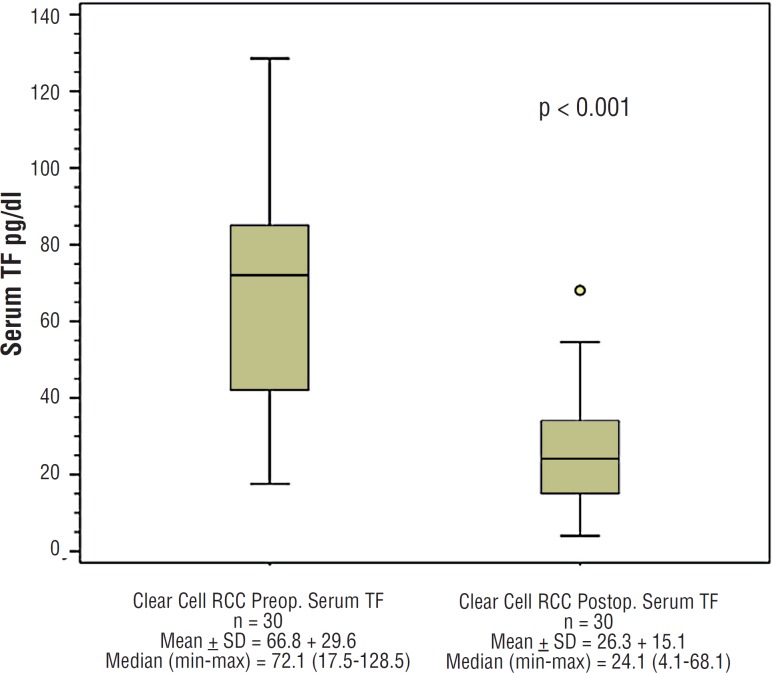
Preoperativa and Postoperative serum TF.

Figure-2 shows postoperative serum levels of TF in patients with clear cell RCC, whose mean was 26.3pg/dL. All patients with clear cell RCC presented with a reduction in serum TF levels after at least four weeks from surgery. The mean reduction in postoperative TF was 41.6pg/dL (p value, 0.001).

To determine which factors are involved in the postoperative reduction of TF, we performed a linear regression for continuous variables using the tumor size data and a covariance analysis for the TNM, presence of necrosis, and Fuhrman grade (Table-2). Tumor size was the only variable that in linear regression showed statistical significance with a value b (Pearson coefficient) of -382 and an angular coefficient (linear regression) of -4.72. This means that with each 1cm increase in tumor diameter corresponds to a reduction of approximately 4.7TF units in pg/dL (p=0.037). The value of preoperative TF was also correlated with the size of the tumor at diagnosis. The Pearson test also confirmed a positive correlation with p=0.03 and an angular coefficient of -388.

**Table 2:** Association between the decrease in serum TF levels and selected prognostic factors.

Characteristics		n	Statistics	p
**Tumor size, cm**		30	r = -0.382	0.03
			b = -4.73	
**Necrosis**				
	Absent	24	−37.8±6.7	0.20
	Present	6	−56.8±11.6	
**Fuhrman grade**				
	1	4	−68.9±11.0	0.07
	≥2	26	−37.4±6.3	
**TNM stage**				
	I	22	−38.1±7.0	0.32
	≥II	8	−51.5±31.1	

**r =** Pearson coefficient; **b =** angular coefficient (linear regression); **p =** statistical significance.

± Sign represents mean ± standard error.

## DISCUSSION

In some malignant neoplasms, serum markers are important not only for the diagnosis of the disease but also have roles as prognostic tools and are adjuncts to patient’s follow-up. So far, no serum marker has proven to be clinically useful in clear cell RCC. We have shown for the first time that in the preoperative setting, patients with clear cell RCC have serum TF levels three times higher than those of patients with other diseases. Our control Group consisted of a very heterogeneous group including one patient with prostate cancer and two patients with benign renal lesions. It is interesting to note that even with a few patients in the control Group presenting with benign neoplasms, the value of preoperative TF in the control Group did not approach the values of patients with ccRCC. We could hypothesize that TF is a more specific endothelial marker of malignant disease, especially in more vascularized organs, like the kidney. Moreover, we have shown a marked (three-fold) reduction in serum TF levels in the postoperative setting of patients with clear cell RCC after at least four weeks of the surgical removal of the tumor. These findings suggest that serum levels of TF may be clinically useful as markers of the presence of clear cell RCC.

The idea of testing this marker in the serum of patients with clear cell RCC was not random. We have previously studied the immunohistochemical expression of TF in tumor samples of patients with Wilms tumors and in tumor samples of patients with clear cell RCC. In the samples of patients with Wilms tumors, an increased TF expression was an independent prognostic factor of overall survival (HR 5.6; p<0.01) (20). In samples of patients with clear cell RCC an increased expression of TF was also an independent predictor of overall survival (HR 4.03; p=0.03) (21). Förster et al. have previously studied TF both in tissue and in serum of patients with RCC. These authors did not find an increased expression of TF in the tumor samples of patients with RCC compared with normal tissue samples of the same kidney. Additionally, they did not find a statistical significant difference in the serum TF levels of patients with RCC and healthy volunteers (22). The reason for these discordant results remains to be explained.

We have observed a marked reduction of serum levels of TF in the postoperative setting in all our patients with clear cell RCC, with a mean reduction of 41pg/dL (p <0.001). Tumor size was correlated with the reduction in serum TF levels, in such a way that for every centimeter of the primary lesion there was a mean reduction of 4.72pg/dL in serum TF in the postoperative setting. Other prognostic variables such as TNM, Fuhrman grade, and the presence of necrosis were tested by covariance analysis, but were not significantly associated with the decrease in TF levels. Our small sample and the predominance of patients with initial disease (e.g., TNM I=73.3%; 20% of SRM's-tumors smaller than 3.5cm) may have contributed to these findings.

Serum TF has been studied in clinical research. The ELISA technique is the most common methodology of serum TF testing (American Diagnostic and R&D). These kits refer to normal levels in healthy patient’s values ranging from 27 to 172pg/mL. Han et al. have previously studied serum TF levels in women with ovarian benign lesions, ovarian lesions of uncertain behavior and ovarian cancer. In this study, mean serum TF levels were of 85.2pg/mL in ovarian cancer, a value much higher of the mean 12.8pg/mL found in patients with benign ovarian diseases (p<0.01). Values higher than 190pg/mL were correlated with a worse prognosis and cancer specific mortality (3-4 times higher; p=0.01) (18). In another study, Zwicker et al. evaluated serum TF with the technique of microparticles (MP-TF) in patients with pancreatic cancer. In this study, there was a correlation between serum MP-TF levels and the risk of thromboembolism and of cancer specific mortality. In three patients, MP-TF was undetectable after the surgical removal of the pancreas (23).

Our study has several limitations. Firstly, we tested a small number of selected patients with clear cell RCC. Larger studies are needed to corroborate our findings. Secondly, since our study was prospective and follow-up is short, we do not have long-term survival or mortality data to evaluate as outcome measures. Thirdly, since cancer determines an inflammatory response, in the presence of a malignant neoplasm there is an increased production of several markers, which may not be cancer specific, but related to inflammation. In this study, we did not perform other inflammatory markers, such as C-reactive protein, Eritrocyte Sedimentation Rate (ESR) (24) or interleukyn-6 (25). This is probably going to be the subject of further research. Lastly, we did not analyze in this study the tissue expression of TF by immunohistochemistry or polymerase-chain reaction techniques in the samples of clear cell RCC.

Our findings should be validated by studies with a larger number of patients to ascertain their real clinical applicability. The usefulness of TF in serum as a marker of the presence of disease could help more accurately to evaluate small renal masses, complex cystic lesions, oncocytomas and angiomiolipomas. Serum TF determinations could also be potentially used as a follow-up tool in clear cell RCC.

## CONCLUSIONS

We have shown a 3-fold reduction in the median preoperative serum levels of TF in patients with clear cell RCC after surgery. We have also shown a difference of the same magnitude in the serum levels of TF compared with those of a control Group of patients with benign diseases. TF appears to be a useful serum marker for the presence of clear cell RCC. Further studies are needed to validate these findings.

## Abbreviations

TF: tissue factor
RCC: renal cell carcinoma
ELISA: enzyme linked immunosorbent assay
